# Supplementary material for: Molehill Mountain feasibility study: Protocol for a non-randomised pilot trial of a novel app-based anxiety intervention for autistic people
Source: PLoS One. 2023 Jul 5;18(7):e0286792. doi: 10.1371/journal.pone.0286792 (PMC10321642; doi:10.1371/journal.pone.0286792)
Supplement: S1 File — S1 Material. Goal attainment scale (Baseline, Endpoint). S2 Material. Study protocol. (DOCX) [file pone.0286792.s002.docx]

**Supplementary Material 1**

**Goal Attainment Scale - Baseline**

**Setting personal goals**

We would like you to set up to 4 goals that you would like to achieve by the end of the study, through using the Molehill Mountain app.

Below, are a list of goals. Please select **up to** 4 goals that are most important to you. We will then ask you to indicate their importance and achievability for you at this time.

At the end of the study, we will ask you for an update on your personal goals. If you think you may not remember which goals you have chosen by then, you may want to make a note of them to remind yourself later.

| **Goals** | **How important is this goal to you?**  1. Not at all important  2. A little important  3. Moderately important  4. Very important | **How far away from achieving this goal do you feel right now?**  1. A long way from this goal  2. A little way from this goal  3. I have achieved this goal  4. I have exceeded this goal  5. I have exceeded this goal by a lot |
| --- | --- | --- |
| 1. I would like to feel less anxious about communicating over the phone (e.g., with professionals, with friends, in front of others, etc) and/or answering the front door |  |  |
| 2. I would like to feel less anxious when myself or somebody else is not following the rules |  |  |
| 3. I would like to feel less anxious about change (e.g., learning new ways to do things, disruptions in routine/plan, when something is out of order or out of place, etc) |  |  |
| 4. I would like to feel less anxious about different sensory environments or triggers (e.g., lots of lights or music, screaming children, loud noises, multiple people talking, etc) |  |  |
| 5. I would like to feel less anxious about social situations, being out in public, communicating, and talking to others |  |  |
| 6. I would like to feel less anxious when meeting new people or making new friends |  |  |
| 7. I would like to feel more confident to mask less and be myself in social situations (e.g., speaking more about my interests). |  |  |
| 8. I would like to be more assertive and to feel less anxious about speaking up in social situations, such as asking questions and giving an opinion. |  |  |
| 9. I would like to feel less anxious in my relationships (e.g., with friends, family, partner) |  |  |
| 10. I would like to feel less anxious when approaching unknown or new situations |  |  |
| 11. I would like to feel more motivated to try new things and/or to do the things I know will help me |  |  |
| 12. I would like to feel less anxious about starting activities, and/or stopping them before finishing |  |  |
| 13. I would like to feel less anxious about making decisions and/or planning activities |  |  |
| 14. I would like to feel less anxious about not doing well at something, when I don’t understand things or when I make a mistake |  |  |
| 15. I would like to feel less anxious about meeting other people’s expectations |  |  |
| 16. I would like to understand my anxiety, my triggers and myself more. |  |  |
| 17. I would like to develop some coping strategies to help with my worries and my anxiety (e.g. talking to others about my anxiety, etc). |  |  |
| 18. I would like to compare myself to neurotypical people less |  |  |
| 19. I would like to worry less about things that have happened in the past |  |  |
| 20. I would like to feel less anxious in general |  |  |

**Goal Attainment Scale - Endpoint**

**Your personal goals**

Earlier in the study, we asked you to set up to **4** personal goals that you would like to achieve through using the Molehill Mountain app. You may have kept a copy of these goals to remind yourself.

We will now ask some questions about your goals.

| **Goals [branching logic so participant only sees their selected 4 goals]** | **How far away from achieving this goal do you feel right now?**  1. A long way from this goal  2. A little way from this goal  3. I have achieved this goal  4. I have exceeded this goal  5. I have exceeded this goal by a lot |
| --- | --- |
| 1. I would like to feel less anxious about communicating over the phone (e.g. with professionals, with friends, in front of others, etc) and/or answering the front door |  |
| 2. I would like to feel less anxious when myself or somebody else is not following the rules |  |
| 3. I would like to feel less anxious about change (e.g. learning new ways to do things, disruptions in routine/plan, when something is out of order or out of place, etc) |  |
| 4. I would like to feel less anxious about different sensory environments or triggers (e.g. lots of lights or music, screaming children, loud noises, multiple people talking, etc) |  |
| 5. I would like to feel less anxious about social situations, being out in public, communicating, and talking to others |  |
| 6. I would like to feel less anxious when meeting new people or making new friends |  |
| 7. I would like to feel more confident to mask less and be myself in social situations (e.g. speaking more about my special interests). |  |
| 8. I would like to be more assertive and to feel less anxious about speaking up in social situations, such as asking questions and giving an opinion. |  |
| 9. I would like to feel less anxious in my relationships (e.g. with friends, family, partner) |  |
| 10. I would like to feel less anxious when approaching unknown or new situations |  |
| 11. I would like to feel more motivated to try new things and/or to do the things I know will help me |  |
| 12. I would like to feel less anxious about starting activities, and /or stopping them before finishing them |  |
| 13. I would like to feel less anxious about making decisions and/or planning activities |  |
| 14. I would like to feel less anxious about not doing well at something, when I don’t understand things or when I make a mistake |  |
| 15. I would like to feel less anxious about meeting other people’s expectations |  |
| 16. I would like to understand my anxiety, my triggers and myself more. |  |
| 17. I would like to develop some coping strategies to help with my worries and my anxiety (e.g. talking to others about my anxiety, etc). |  |
| 18. I would like to compare myself to neurotypical people less |  |
| 19. I would like to worry less about things that have happened in the past |  |
| 20. I would like to feel less anxious in general |  |

Thank you for taking the time to share this with us.

**Supplementary Material 2**

1. **Feasibility study of an adapted app-based anxiety intervention for autistic people.**

Trial Identifiers

| **ClinicalTrials.gov:** | Record 1286908 [[Molehill Mountain Feasibility Study. - Full Text View - ClinicalTrials.gov](https://clinicaltrials.gov/ct2/show/NCT05302167)] | | |
| --- | --- | --- | --- |
| **REC Number:** | IRAS 308723; 22/LO/0291 | | |
| **Protocol Version Number:** | 2 | Date: | 18.08.2022 |

(Co) Sponsor(s)

*This is usually the substantive employer of the CI. If this is KCL then co-sponsorship with the participating NHS Trust may be required.*

| **Name (Lead sponsor):** | King’s College London |
| --- | --- |
| **Address:** | Room 5.31, James Clerk Maxwell Building, 57 Waterloo Road, London, SE1 8WA |
| **Telephone:** | +44 (0)207 848 3224 |
| **Email:** | reza.razavi@kcl.ac.uk |

| **Name (Co-sponsor):** | South London and Maudsley NHS Foundation Trust |
| --- | --- |
| **Address:** | R&D Department, Room W1.08, Institute of Psychiatry, Psychology & Neuroscience (IoPPN), 16 de Crespigny Park, London, SE5 8AF |
| **Telephone:** | +44 (0)207 848 0339 |
| **Email:** | slam-ioppn.research@kcl.ac.uk |

Chief Investigators

| **Name:** | Prof Emily Simonoff |
| --- | --- |
| **Address:** | Department of Child and Adolescent Psychiatry  Institute of Psychiatry, Psychology and Neuroscience  16 de Crespigny Park  Camberwell  London  SE5 8AF |
| **Telephone:** | +44 (0)207 848 5312 |
| **Email:** | emily.simonoff@kcl.ac.uk |

**Name and address of Co-Investigator(s), Statistician, Therapy Service, Laboratories etc**

| **Name:** | Dr Bethany Oakley |
| --- | --- |
| **Position/ Role:** | Postdoctoral Research Associate |
| **Address:** | Department of Forensic and Neurodevelopmental Sciences  Main IOPPN Building  16 de Crespigny Park  Camberwell  London  SE5 8AF |
| **Telephone:** | +44 (0)207 848 0820 |
| **Email:** | bethany.oakley@kcl.ac.uk |

| **Name:** | Miss Charlotte Boatman |
| --- | --- |
| **Position/ Role:** | Research Assistant |
| **Address:** | Department of Forensic and Neurodevelopmental Sciences  Main IOPPN Building  16 de Crespigny Park  Camberwell  London  SE5 8AF |
| **Telephone:** | +44 (0)207 848 0820 |
| **Email:** | charlotte.boatman@kcl.ac.uk |

| **Name:** | Prof Declan Murphy |
| --- | --- |
| **Position/ Role:** | Professor of Translational Neurodevelopment, and Director of the Sackler Institute of Translational Neurodevelopment |
| **Address:** | Department of Forensic and Neurodevelopmental Sciences  Main IOPPN Building  16 de Crespigny Park  Camberwell  London  SE5 8AF |
| **Telephone:** | +44 (0)207 848 0982 |
| **Email:** | Declan.murphy@kcl.ac.uk |

| **Name:** | Dr Sophie Doswell |
| --- | --- |
| **Position/ Role:** | Consultant Clinical Psychologist |
| **Address:** | ADHD & ASD Services, NAAAPS  Behavioural & Developmental Psychiatry  Monks Orchard House, Bethlem Royal Hospital  Beckenham, Kent BR3 3BX |
| **Email:** | sophie.doswell@slam.nhs.uk |

| **Name:** | Dr Antonia Dittner |
| --- | --- |
| **Position/ Role:** | Principal Clinical Psychologist |
| **Address:** | ADHD & ASD Services, NAAAPS  Behavioural & Developmental Psychiatry  Monks Orchard House, Bethlem Royal Hospital  Beckenham, Kent BR3 3BX |
| **Telephone:** | +44 (0)203 228 6171 |
| **Email:** | antonia.dittner@slam.nhs.uk |

| **Name:** | Ms Abigail Oliver |
| --- | --- |
| **Position/ Role:** | Assistant Psychologist |
| **Address:** | ADHD & ASD Services, NAAAPS  Behavioural & Developmental Psychiatry  Monks Orchard House, Bethlem Royal Hospital  Beckenham, Kent BR3 3BX |
| **Telephone:** | +44 (0)203 228 6171 |
| **Email:** | abigail.oliver@slam.nhs.uk |

| **Name:** | Dr Rachel Kent |
| --- | --- |
| **Position/ Role:** | Senior Clinical Psychologist |
| **Address:** | South London and Maudsley NHS Foundation Trust  Michael Rutter Centre for Children & Young People  Maudsley Hospital  De Crespigny Park  London SE5 8AZ |
| **Telephone:** | +44 (0)20 3228 2547 |
| **Email:** | rachel.kent@slam.nhs.uk |

| **Name:** | Dr Ann Ozsivadjian |
| --- | --- |
| **Position/ Role:** | Principal Clinical Psychologist |
| **Address:** | Department of Child and Adolescent Psychiatry  Institute of Psychiatry, Psychology and Neuroscience  16 de Crespigny Park  Camberwell  London  SE5 8AF |
| **Email:** | drann@drannozsivadjian.co.uk |

| **Name:** | Mr Andrew Clarke |
| --- | --- |
| **Position/ Role:** | Product Owner, Molehill Mountain |
| **Address:** | Autistica CAN Mezzanine,  7-12 Great Dover Street,  London SE1 4YR |
| **Email:** | Andrew.Clarke@autistica.org.uk |

1. **Study Synopsis**

| **Title Of Clinical Trial:** | Feasibility study of an adapted app-based anxiety intervention for autistic people. |
| --- | --- |
| **Protocol Short Title/ Acronym:** | Molehill Mountain feasibility study |
| **Study Phase If Not Mentioned In Title:** | Feasibility/ acceptability study |
| **Sponsor Name:** | King’s College London/ South London and Maudsley NHS Foundation Trust |
| **Chief Investigators:** | Prof Emily Simonoff |
| **REC Number:** | 22/LO/0291 |
| **Medical Condition Or Disease Under Investigation:** | Co-occurring anxiety in autism |
| **Purpose Of Clinical Trial:** | To provide an early-stage proof of concept for the feasibility and acceptability of a novel, app-based therapeutic approach (‘Molehill Mountain’) that has been developed with, and adapted for, autistic people to support anxiety management using National Institute for Health and Care Excellence (NICE) recommended adapted CBT approaches. |
| **Primary Objective:** | To establish the acceptability, feasibility and useability of a novel app-based anxiety intervention, specifically developed with and for autistic people (‘Molehill Mountain’), in a real-world clinical setting. |
| **Secondary Objective(s):** | To establish the target population, performance of selected outcome measures and ideal timing/ duration of intervention to inform the design of a future randomised-controlled trial. |
| **Trial Design:** | Other clinical trial to study a novel intervention (feasibility/ acceptability phase) |
| **Endpoints:** | We will establish app acceptability, feasibility and useability (Objective One) via app usage data and an in-depth post-study app use experience survey (all participants)/ semi-structured qualitative interview on app use experience (subsample of participants).  Primary clinical endpoints:   - 7-item Generalised Anxiety Disorder Assessment (GAD-7; Spitzer, Kroenke, Williams and Löwe, 2006). - Hospital Anxiety and Depression Scale (HADS; Zigmond and Snaith, 1983). - App acceptability/ feasibility survey/ interview.   Secondary clinical endpoints:   - Individualised functional outcome, assessed via medication/ service use and Goal Attainment Scale (Turner-Stokes and Williams, 2010). |
| **Sample Size:** | Approximately 200 participants aged ≥16-years with an existing autism diagnosis and mild-to-severe anxiety symptoms. |
| **Summary Of Eligibility Criteria:** | Inclusion criteria:   - Age ≥ 16-years. - Autism diagnosis. - Current mild-to-severe anxiety symptom severity, as assessed at screening. - Able and willing to provide verbal and written informed consent to take part in the study. Fluency in verbal/ written English for the purposes of engaging with the app materials. - Able and willing to use a mobile phone app.   Exclusion criteria:   - Difficulties with reading/ writing to the extent that the app is inaccessible (i.e., able to use the mobile phone app). - High risk of self-harm that make participation in the study inappropriate for the individual’s current level of clinical need (as assessed by clinical team). - Attended ≥ 6 sessions of individual or group therapy (e.g., cognitive behavioural therapy) in the past 6-months, which would make it impossible to parse out the effects of the app from existing therapy. - If using psychotropic medication, this medication/ dose must have been stable for a minimum of 8-weeks on entry to the study. |
| **Intervention (Description, frequency, details of delivery)** | The active investigational app-based intervention (‘Molehill Mountain’, current Version 2) has been developed jointly by King’s College London and UK autism charity Autistica for use by autistic people aged ≥12-years (<https://www.autistica.org.uk/molehill-mountain>).  The Molehill Mountain app has been designed based on Cognitive Behavioural Therapy (CBT) techniques adapted for autism – supporting app-users to recognise and understand triggers for anxiety and incorporating tips and exercises (e.g., mindfulness techniques) to manage anxiety.  The app is self-guided for use with a SmartPhone, or other Smart device (i.e., does not require clinician input after initial setup) and designed to be used over a period of 3-months, with short daily logins. |
| **Comparator Intervention:** | N/A – feasibility/ acceptability study with no randomisation. |
| **Maximum Duration Of Treatment Of A Subject:** | 3-months. |
| **Version And Date Of Final Protocol:** | Version 2, 18.08.2022 |
| **Version And Date Of Protocol Amendments:** | Version 1.1 18.05.2022  Version 2 18.08.2022 |

1. **Revision History**

| **Document ID - (Document Title) revision X.Y** | **Description of changes from previous revision** | **Effective Date** |
| --- | --- | --- |
| Protocol Version 2 18.08.2022 | Addition of details of an invitation letter for prospective participants recruited via SLaM clinics where their preferences regarding being contacted about research studies are currently unknown (i.e., they have neither provided consent, nor disagreed, to be contacted via 'consent for contact'; c4c) to invite them to opt in to hear more about the study.  Update of schedule of events – GAD-7 administered at screening/ enrolment timepoint to assess eligibility (also acting as baseline primary anxiety measure).  Addition of a measure of autistic traits as a covariate (assessed at baseline and endpoint, selected by advisory group).  Addition of research team member (Charlotte Boatman) contributing to protocol amendment. | 17.11.2022 |

1. **Glossary of terms**

AE – Adverse Event

AR – Adverse Reaction

CBT – Cognitive Behavioural Therapy

CI – Chief Investigator

CONSORT – Consolidated Standards of Reporting Trials

CRF – Case Report File

CTIMP – Clinical Trial of an Investigational Medicinal Product

GCP – Good Clinical Practice

GSTT – Guys and St Thomas’ NHS Foundation Trust

KCH – King’s College Hospital

KCL – King’s College London

NHS – National Health Service

REC – Research Ethics Committee

RCT – Randomised controlled trial

SAE – Serious Adverse Event

SAR – Serious Adverse Reaction

SLaM – South London and Maudsley NHS Foundation Trust

SOP – Standard Operating Procedure

SUSAR – Suspected Unexpected Serious Adverse Reaction

TMG – Trial Management Committee

UAR – Unexpected Adverse Reaction

1. **Protocol Contents**

**1.** **Feasibility study of an adapted app-based anxiety intervention for autistic people. 1**

**2.** **Study Synopsis 5**

**3.** **Revision History 7**

**4.** **Glossary of terms 7**

**5.** **Protocol Contents 8**

**6.** **Background & Rationale 10**

7.1 Trial Objectives 11

7.1.1 Primary endpoints 11

7.1.2. Secondary endpoints 11

7.2 Trial Design 12

7.3 Trial Flowchart 12

**8** **Trial Intervention 14**

8.1 Therapy/Intervention Details 14

8.2 Frequency and duration of intervention 15

8.3 Intervention records 15

8.4 Participant adherence. 15

8.5 Concomitant Medication 15

**9** **Research environment 15**

**10** **Selection and Withdrawal of Subjects 16**

10.1 Inclusion Criteria 16

10.2 Exclusion Criteria 17

10.3 Withdrawal of Subjects 17

10.4 Expected Duration of Trial 17

**11** **Trial Procedures 17**

11.1 By Visit 17

**12** **Assessment of Effectiveness 20**

**13** **Assessment of Safety 21**

13.1 Specification, Timing and Recording of Safety Parameters 21

13.2 Procedures for Recording and Reporting Adverse Events 21

*13.2.1* *Adverse events that do not require reporting 22*

13.3 Stopping Rules 23

**14** **Statistics 23**

**16** **Direct Access to Source Data and Documents 24**

**17** **Ethics & Regulatory Approvals 24**

**18** **Quality Assurance 24**

**19** **Data Handling and Management 24**

**20** **Publication Policy 25**

**21** **Insurance / Indemnity 25**

**22** **References 25**

**23** **Appendixes 26**

1. **Background & Rationale**

***Background***

Autism is a lifelong neurodevelopmental condition that affects ~1% of the population and is characterised by social-communication difficulties and restricted and repetitive behaviours (including sensory processing differences; American Psychiatric Association, 2013). Up to 70% of autistic people experience co-occurring mental health problems (e.g., anxiety; Simonoff et al., 2008; Hollocks et al, 2019), which contribute significantly to reduced quality of life (Mason et al., 2019; Oakley et al., 2020; van Steensel, Bögels, & Perrin, 2011), increased caregiver burden (Cadman et al., 2012) and suicide - the leading cause of premature mortality for autistic people without intellectual disability (Hirvikoski et al., 2016). Thus, developing new interventions (and/ or adapting existing ones) that improve anxiety represents the autism community’s number one priority for research and clinical practice (Autistica, 2017).

However, there are very few effective, evidence-based interventions to support autistic people in managing anxiety (White et al., 2018). There are no pharmacological treatments for anxiety currently approved by regulators for use in autism. Recommendations for the use of selective serotonin reuptake inhibitors (SSRIs; e.g., sertraline, fluoxetine) to manage anxiety in autism are predominantly based on evidence from typically developing groups and associated with elevated side effect profiles (Howes et al., 2018; Williams and Hazell, 2013).

Similarly, though mindfulness and adapted CBT approaches have been identified as emerging evidence-based therapies for anxiety in autism (Benevides et al., 2020) and are recommended by the National Institute of Health and Care Excellence, access to adapted CBT remains limited due to severe pressures on clinical services.

Directly responding to this high unmet need, the primary objective of the current study is to establish the acceptability and feasibility of a novel app-based anxiety intervention, specifically developed with and for autistic people and incorporating NICE recommended adapted CBT principles (‘Molehill Mountain’), in a real-world clinical setting.

***Molehill Mountain***

The Molehill Mountain app (currently Version 2) was developed jointly by King’s College London (Lead: Prof Emily Simonoff; Advisors: Dr Ann Ozsivadjian and Dr Rachel Kent) and UK autism charity Autistica (Product Owner: Andy Clarke; Product Designer: Joanna Alpe) for use by autistic people aged ≥12-years (<https://www.autistica.org.uk/molehill-mountain>).

The app was designed based on a self-guided paper-and-pencil CBT toolkit adapted for autism by Professor Emily Simonoff; and is now available to SmartPhone users at no charge. Since the first version of Molehill Mountain (a two-week long programme) was launched in 2016, and based on surveys of autistic people and five rounds of prototype testing, the Molehill Mountain V2 app was relaunched in April 2021 as a three-month long app-based programme with enhanced interactive features. It is this 3-month V2 Molehill Mountain app, with enhanced interactive features supporting short daily logins, that will be implemented in the current study.

An investigational active app-based intervention specifically adapted for autistic people (vs. an intervention developed in the wider population) was deemed to be most suitable for the proposed project, based on existing best practice guidance on autism-specific modifications to psychological/ psychosocial therapies (e.g., incorporating concrete tools and support, addressing core autism features, incorporation of special interests; Cooper, Loades, & Russell, 2018; National Institute of Health and Care Excellence, 2013). In addition, the choice of an app-based intervention (vs. in-person therapy) was driven by consideration of both potential cost-effectiveness and the significant barriers that many autistic people experience in accessing existing health and social care services, including individual differences in communication needs and sensory sensitivities (Mason et al., 2019; Oakley et al., 2021).

Novel digital approaches for delivering intervention, such as Molehill Mountain, may address some of these barriers to service access in a way that also responds to the current need for increased flexibility in health and social care delivery, as emphasised by the SARS-CoV-2 pandemic. Molehill Moutain has potential to provide further added value in terms of: a) cost, with app-based interventions having potential to reduce financial costs to services, and improve access/ reduce waiting times to support (NHS England, 2019); b) time/ intensity, for both the participant who can utilise the app intervention flexibly at any time and in any place, as well as the clinical team who only provide minimal input after initial setup; c) availability, with 98% of the target population estimated to have access to a SmartPhone and Molehill Mountain compatible for use with both Apple and Android phones; and d) risk, with app-based interventions considered low-risk, as compared to existing pharmacological approaches that are known to be associated with elevated side effects in autism (Williams and Hazell, 2013).

Nevertheless, despite their high potential, the use of digital tools in mental health service delivery in autism remains strikingly understudied (Sutherland, Trembath and Roberts, 2018). Thus, there are several issues that must be addressed in this research, prior to seeking funding for a full-scale randomised-controlled trial. First, we need to better understand levels of engagement with the app – for instance, the proportion of autistic people in clinical services who are willing to download the app, and, subsequently, the duration of app use and app functions that are feasible/ acceptable for this population. This is essential to identify where app modifications are most needed to enhance inclusivity and engagement. Additionally, we need to further establish the useability and technical functionality of the app. For instance, though Molehill Mountain has already gone through several rounds of development in consultation with autistic people and their families, it has not yet been tested in a clinical population and further minor modifications may, therefore, be necessary. Last, it is crucial that we gather evidence to optimise a future definitive trial design, including establishing the target population (e.g., age, anxiety severity threshold), selection of key clinical outcome measures, and ideal timing for endpoint assessment.

1. **Trial Objectives and Design**
   1. ***Trial Objectives***

To support these essential next steps, we set out two overarching objectives for the proposed study:

***Objective One:*** To establish the acceptability, feasibility and useability of a novel app-based anxiety intervention, specifically developed with and for autistic people (‘Molehill Mountain’), in a real-world clinical setting.

***Objective Two:*** To establish the target population, performance of selected outcome measures and ideal timing/ duration of intervention to inform the design of a future randomised-controlled trial.

- 1. *Primary endpoints*

The primary outcome measures in this study will be anxiety symptom severity, as assessed using the 7-item Generalised Anxiety Disorder Assessment (GAD-7; Spitzer, Kroenke, Williams and Löwe, 2006); and the Hospital Anxiety and Depression Scale (HADS; Zigmond and Snaith,1983). Including the GAD-7 and HADS will allow us to test the performance of a candidate outcome measure for a future trial (Objective Two), based on assessment tools already being used in SLaM clinical services and commonly used intervention studies with the target population.

*ii. Secondary endpoints*

In addition, secondary outcome measures will include individualised functional outcome (assessed via medication/ service use and Goal Attainment Scale; Turner-Stokes and Williams, 2010), which is both meaningful to the target population (Oakley et al., 2020) and may be more sensitive in reflecting subtle improvements following intervention than standardised symptom scales like the GAD-7/ HADS.

*iii. Covariates*

Last, a measure of autistic traits will be included as a covariate to be considered alongside demographic features. This is to ascertain whether potential change in anxiety for some participants might be associated with baseline demographic factors and/ or autistic traits (i.e., whether factors aside from the intervention might predict change in anxiety over the course of the study, and if so, which ones).

Two existing validated measures of autistic traits are included: the Adult Routines Inventory (Evans, Uljarevic, Lusk, Loth and Frazier, 2017); and the Comprehensive Autistic Trait Inventory (English, Gignac, Visser, Whitehouse, Enns and Maybery, 2021). These two measures were preferred by the Molehill Mountain advisory group from a selection of commonly used measures of autistic traits (also including the AQ and SRS-2), and will be integrated into the baseline and endpoint assessment.

- 1. ***Trial Design***

An early-stage proof of concept design to assess the feasibility and acceptability of a novel, app-based therapeutic approach (‘Molehill Mountain’) that has been developed with, and adapted for, autistic people to support them in managing anxiety using National Institute for Health and Care Excellence (NICE) recommended adapted CBT approaches.

- 1. ***Trial Flowchart***

Note: We will aim for a flexibility of +/- 2-weeks for trial procedures from enrolment to endpoint assessment, and +/- 4-weeks in the follow-up assessment period. However, if this is not possible, we will still collect data outside of these windows and clearly report this.

Week 0 – Screening for eligibility/ enrolment: Participants are contacted to ascertain interest and assess eligibility for the study through a brief screening process based on inclusion/ exclusion criteria. All participants are consented, so that basic demographic data can be collected to compare enrolled vs. non-enrolled participants. Interested and eligible participants are fully consented and enrolled into the study. Participants who decline are invited for a brief post-study ‘exit’ survey/ interview to understand reasons for decline.

Week 2 (+/- 2) – Baseline assessment and app administration: Enrolled participants complete primary/ secondary endpoints as a baseline assessment (please also see Table 1, below). The clinical research team will support those enrolled to download and initiate the Molehill Mountain app to a SmartPhone or similar device and provide a brief introduction/ participant pack on using the app.

Weeks 2-15 (+/- 2) – Intervention period: Participants undergoing 3-month intervention period, with a remote check-in at 8-weeks to monitor engagement, respond to queries/ concerns, monitor adverse events. This does not preclude participants from contacting the research team before/ after the 8-week check in period – for instance, if they are having problems to use the app.

Week 15 (+/- 2) – Endpoint assessment: Participants and clinical team invited to complete post-study survey and interview; primary/ secondary endpoints re-assessed (please also see Table 1, below). Where interviews take place remotely, these will be held via video call using Microsoft Teams wherever possible.

Week 24 (+/- 4) – Follow-up assessment 1: Primary/ secondary endpoints re-assessed (please also see Table 1, below).

Week 32 (+/- 4) – Follow-up assessment 2: Primary/ secondary endpoints re-assessed (please also see Table 1, below).

Week 41 (+/- 4) – Follow-up assessment 3: Primary/ secondary endpoints re-assessed (please also see Table 1, below).

**Table 1. *Schedule of Activities***

|  | **Screen/ enrol** | **Baseline** | **Intervention** | **Endpoint** | **F/up 1** | **F/up 2** | **F/up 3** |
| --- | --- | --- | --- | --- | --- | --- | --- |
| **Week** | 0 | 2  (+/-2) | 2-15  (+/-2) | 15  (+/-2) | 24 (+/-4) | 32 (+/-4) | 41 (+/-4) |
| **Information sheet** | X |  |  |  |  |  |  |
| **Consent form** | X |  |  |  |  |  |  |
| **Screening (Eligibility check)** | X |  |  |  |  |  |  |
| **Demographics** | X |  |  |  |  |  |  |
| **Exit survey/ interview*** | X |  |  |  |  |  |  |
| **GAD-7^1^** | X** |  |  | X | X | X | X |
| **HADS^2^** |  | X |  | X | X | X | X |
| **Goal Attainment Scaling^2^** |  | X |  | X | X | X | X |
| **Medication/ service use questionnaire^2^** |  | X |  | X | X | X | X |
| **Autistic traits (ARI, CAT-I)** |  | X |  | X |  |  |  |
| **App administration** |  |  | X |  |  |  |  |
| **Remote contact/ check-in** |  |  | X  (Week 8) |  |  |  |  |
| **App acceptability survey^1^/ interview** |  |  |  | X |  |  |  |
| **Adverse events** |  | | | | | | |

Note: F/up = Follow-up; GAD-7 = 7-item Generalised Anxiety Disorder scale; HADS= Hospital Anxiety and Depression Scale; ARI = Adult Routines Inventory; CAT-I = Comprehensive Autistic Trait Inventory (CAT-I). ^1^Outcome measures of highest priority (I.e., if the participant is only able to complete one measure it would be this one); ^2^Outcome measures of secondary priority. *Only for those who decline/ are not enrolled; **To establish eligibility criteria of mild-to-moderate anxiety symptom severity – also acting as a baseline primary anxiety measure.

1. **Trial Intervention**
   1. ***Therapy/Intervention Details***

*8.1.1 Active app intervention:*

Molehill Mountain V2 consists of six key components to support app users to become more aware of the triggers and protective factors associated with their anxiety and begin to incorporate strategies to manage anxiety (Figure 1).


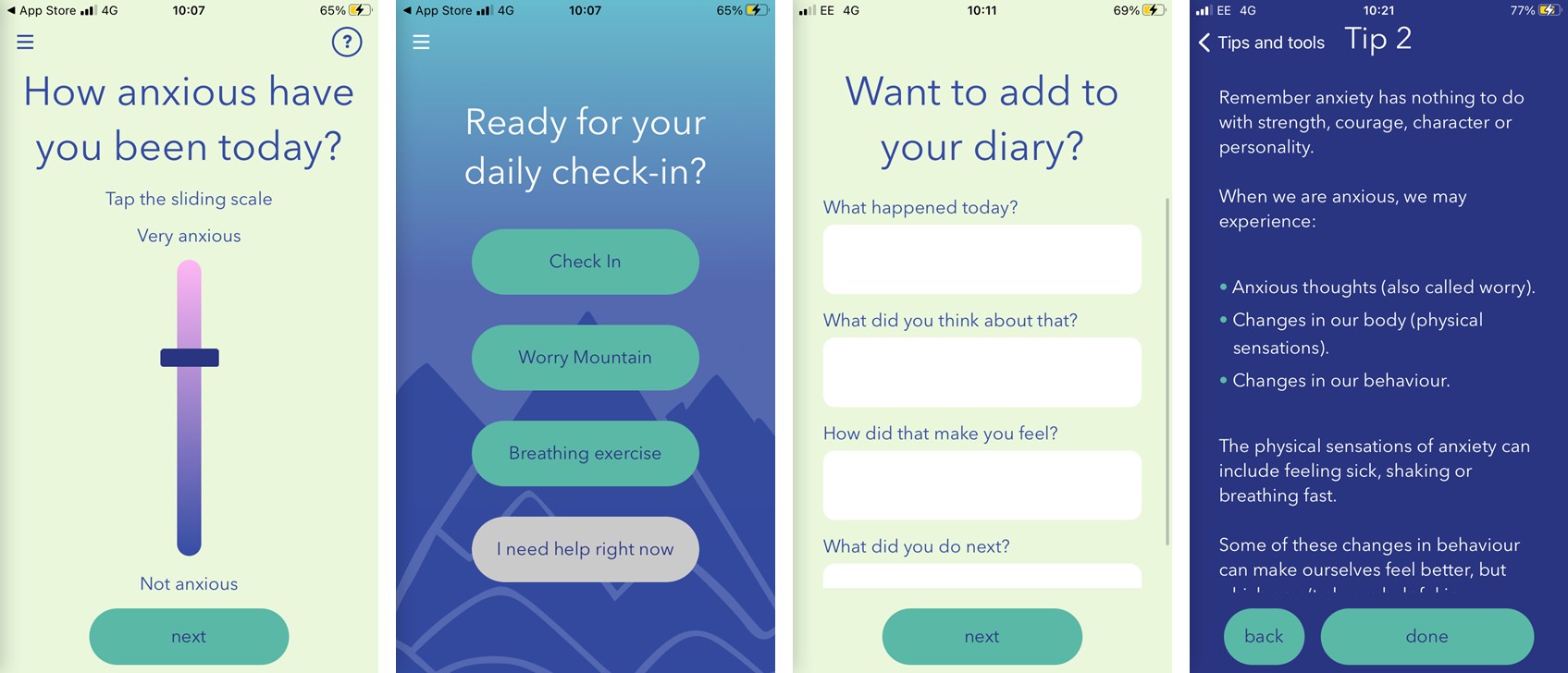


*Figure 1.* Examples of Molehill Mountain app functionality. From left to right: Daily anxiety severity sliding scale; Daily check-in (reporting worries, relaxation techniques); Daily diary of activities and feelings; Anxiety tips and tools.

Firstly, app users can ‘check in’ daily to report how anxious they have felt today (on a sliding scale from ‘not anxious’ to ‘very anxious’), what they have been worried about today (e.g., ‘meeting new people’, ‘health’ etc.), what they did today (e.g., ‘had a walk’), as well as a more in-depth daily diary of experiences and feelings. From time to time, app users are prompted to complete a very short anxiety questionnaire.

Second, app users can view their progress using a drop-down menu in the homepage of the app – with statistics provided on how many days in a row they have checked in to the app, a mood tracker of their self-reported anxiety, a worry count to view how self-reported worries have changed over time, and an in-depth daily diary of experiences and emotions.

The Molehill Mountain app additionally includes two low-intensity anxiety management exercises that app users can engage with. For instance, in the ‘Worry Mountain’ game, participants can unlock their worries for 30 seconds, which then appear as floating balloons that can be popped, before being locked away again. Further to this, the app includes a ‘Breathing Exercises’ page, which prompts participants to engage in a calming breathing technique by breathing in for 4 seconds, holding for 4 seconds, breathing out for 4 seconds, and holding for 4 seconds, for a total cycle of 120-seconds.

Another core component of the app is the ‘Tips and Tools’ section, which allows app users to unlock various strategies for understanding and better managing anxiety, based on CBT principles. Each time a new tip/ tool is unlocked, participants receive encouragement (e.g., ‘keep on going!’ for their continued engagement with the app.

Finally, there is a location in the app for emergencies (‘I need help right now’), which signposts app users to support services (e.g., the Samaritans), and also provides the option for app users to log their own emergency contact (e.g., a relative, friend, support worker etc.).

- 1. ***Frequency and duration of intervention***

The intervention duration will be 3-months, with participants being encouraged to engage with the app daily (e.g., once per day). However, the intervention is completely self-guided by the participant, who will have autonomy over when, where and for how long they engage with the app. Please see Table 1 for a detailed overview of the duration of participation, from first contact to follow-up 3.

- 1. ***Intervention records***

All parts of the study are designed to be completed remotely (e.g., online, by telephone, email, or by post). To facilitate this, all study documentation will be made available via online survey and in paper-and-pencil format. If preferred by the participant (and feasible), some aspects of the study (e.g., informed consent, exit/ post-study interviews) can take place in person.All contact with participants, whether this takes place remotely or in person, will be recorded in the participants’ unique Case Report File (CRF). Engagement with the intervention itself is recorded via the app.

REDCap e-data management system will be used as the database for pseudonymised participant data. Access to this platform will be restricted to the research team.

- 1. ***Participant adherence.***

To encourage study adherence, the research team will provide participants with a brief overview and a participant pack detailing the full study protocol in an easy access manner. As part of these materials, participants will be encouraged to briefly engage with the app daily for 3-months. This will be reiterated at the Week 8 telephone check in, where clinical research team can discuss with participants their app usage and provide any feedback on participant queries/ concerns. The study protocol includes regular follow-up assessments (~every 2-months), where ability to contact participants will also indicate adherence with the study procedures. The app itself can also help the research team to monitor participant adherence, as it provides an internal record of logins that can be used to index regularity of app usage. Therefore, we will ask participants to keep app usage monitoring turned on, however we will not make agreement to this an inclusion criterion and participants will have the option to switch this function off if they choose to (also providing information on the acceptability of app usage monitoring for a full scale trial).

However, as noted earlier, the intervention is completely self-guided by the participant, who will have autonomy over when, where and for how long they engage with the app. Consequently, it will not be possible to always ensure study adherence. Nevertheless, one of the primary aims of the study is to assess the acceptability/ feasibility of the intervention in its current form. Therefore, non-adherence to the study protocol will be a key indicator of potential adjustments that may be required to the app and its format to facilitate a future full-scale RCT.

- 1. ***Concomitant Medication***

All forms of concomitant psychotropic medication will be acceptable, so long as the medication and dose has been stable for a minimum of 8 weeks on entry to the study. Accepting participants using medication to the study will ensure that the sample is as representative as possible, given that at least 27% of autistic people will be prescribed at least one form of psychotropic medication at least once in their lifetime (Howes et al., 2018).

However, participants should not be concurrently attending individual or group therapy using techniques that overlap with those of the app-based intervention (e.g., CBT, mindfulness-based therapy), as this would make it impossible to parse out the effects of the app from existing therapy.

1. **Research environment**

***9.1 Selection of participants***

Participants will be recruited, and data collected, via two sources:

1. The primary location for the study will be South London and Maudsley (SLaM) NHS Foundation Trust, a mental health service in the UK. SLaM is part of an academic health sciences centre called King's Health Partners with King’s College London (KCL), Guy’s and St Thomas’ (GSTT) and King’s College Hospital (KCH). Potential participants recruited via clinic services will be identified by clinical teams via: consent for contact (c4c; https://www.slam.nhs.uk/quality-and-research/take-part-in-research/consent-for-contact-c4c/); outpatient clinics that serve those with autism and co-occurring mental health; associated clinical team meetings; recommendation by a clinician responsible for an individual's care that this named individual may be interested in participating. For additional context, consent for contact (or c4c) is a register of people accessing SLaM services who would be willing for researchers to contact them to ask if they would like to take part in current or future research projects that interest them. For prospective participants where their consent preferences for being contacted about research are unknown, the clinic team will send an invitation letter (on a maximum of two occasions) to invite them to contact the research team directly should they be interested to find out more information about the study. Prior to initiating participant recruitment through c4c, the research team will apply for internal approvals within SLaM.
2. Participants may also be recruited, and data will be processed and analysed at KCL, a University in the UK. Potential participants recruited via existing research studies will be identified and approached by the corresponding research team(s), and only if they have previously consented to be contacted about additional research.

As described in further detail in Section 11, much of the protocol is designed to be administered remotely (e.g., online, via telephone, video call or post). This is to minimise both participant and clinician burden, as well as promote inclusion and participation by those who may have greater difficulties accessing in person visits (and thus for whom an app-based intervention approach may be of particular added value).

In some cases, the participant may prefer to complete aspects of the study face-to-face, either via an Institute visit or at home. Where visits take place in person (and particularly at home) there is a small chance that researchers may be exposed to risks, including potential COVID-19 related risks, or encountering aggression. For Institute visits, we will complete a full Departmental risk assessment to ensure that all aspects of the study comply with local regulations. For home visits (which will be conducted in very rare circumstances where participants are unable to complete the study remotely/ via an Institute visit), where possible these will be conducted by researchers in pairs. Where home visits are conducted by a lone researcher, they should only take place during daytime weekday working hours and the researcher must have first had contact with the participant over the phone. As participants are predominantly being recruited via clinic waitlists, their history and circumstances will be known in advance. Researchers conducting home visits should notify the team the exact date/ time of their visit (including the projected end time), and contact the team on arrival and departure. Researchers will be briefed on safe practices in advance of home visits (e.g., to survey safe exit routes on arrival, sit nearest to the door, to have an exit strategy prepared e.g., “I need to get some paperwork from the car.”) For all in-person assessments, researchers will follow local COVID-19 guidance (including mask wearing, participant symptom screening).

This research study is entirely separate to participants’ clinical care and access to usual services and support is in no way affected by involvement in this study. We will seek participant consent to notify their GP of their involvement in the study.

1. **Selection and Withdrawal of Subjects**
   1. ***Inclusion Criteria***

- Age ≥ 16-years.
- Autism diagnosis.
- Current mild-to-severe anxiety symptom severity, as assessed at screening (and currently in touch with mental health services e.g., referred, on the waitlist).
- Able and willing to provide verbal and written informed consent to take part in the study (and to consent to their GP being contacted regarding their involvement in the study). Fluency in verbal/ written English for the purposes of engaging with the app materials.
- Able and willing to use a mobile phone app.
  1. ***Exclusion Criteria***
- Difficulties with reading/ writing to the extent that the app is inaccessible.
- High risk of self-harm that make participation in the study inappropriate for the individual’s current level of clinical need (as assessed by clinical team).
- Attended ≥ 6 sessions of individual or group therapy (e.g., cognitive behavioural therapy) in the past 6-months, which would make it impossible to parse out the effects of the app from existing therapy.
- If using psychotropic medication, this medication/ dose must have been stable for a minimum of 8-weeks on entry to the study.

Participants will predominantly be recruited from clinic wait lists within neurodevelopmental/ mental health services in the UK. Participants may also be recruited via existing databases/ mailing lists at study sites.

- 1. ***Withdrawal of Subjects***

Participants have the right to withdraw from the study at any time for any reason. Participants do not have to provide a reason for withdrawal. If withdrawal from the study is requested, participants will be asked to confirm whether they also wish to withdraw the data that they have provided up to that point, and/ or whether they wish to withdraw from the intervention component but are still happy to be contacted for endpoint assessment/ follow-up timepoints and complete the post-study survey/ interview. However, participants will be made aware during informed consent that pseudononymised basic demographic information and anxiety symptom severity at baseline will be maintained as summary data to enable comparison between the retained study cohort and those withdrawn.

In rarer circumstances, the study team may also recommend that a participant withdraws from the study – for example, as the result of an Adverse or Serious Adverse Event (AE/ SAE). If participants deemed eligible on entry to the study subsequently become ineligible over the course of the study (e.g., because they enter individual/ group therapy), decisions on recommending that the participant withdraws will be taken on a case-by-case basis through study team consultation. For instance, the study team may recommend that a participant withdraws if they enter therapy where the clinical team deem that the study intervention may interfere/ be detrimental to outcome, or where the participants’ anxiety or other mental health needs become so great during the course of the study that the app becomes inappropriate for their clinical need. Nevertheless, as far as possible, forced withdrawal from the study will be minimised so long as remaining in the study is not deemed detrimental to (and by) the participant. All withdrawals will be recorded as thoroughly as possible in the participant CRF.

- 1. ***Expected Duration of Trial***

The start of the trial is defined by the date of the first contact with the first participant. The end of the trial is defined by the date of the final contact at the third and final 41 week (+/-4) follow-up assessment of the last participant randomised. At this stage, declaration of end of study will be made to the REC within 90 days and the database will become locked for analysis.

For each participant, respectively, study duration from first contact to the third and final follow-up assessment will be 41 weeks (+/-2). The end of the trial for each participant is defined by the conclusion of the third and final follow-up assessment at 41 weeks (+/-4; please also see Table 1).

1. **Trial Procedures**
   1. ***By Visit***

Full details of trial procedures at each stage are provided in Table 2. The sequence of events at each stage of the trial, as displayed in Table 2, is also listed in order of prioritisation (e.g., in terms of questionnaire measures at baseline, endpoint and follow-ups).

**Table 2. *Timeline of trial procedures and events.***

| 1. **Screening/ enrolment (Week 0)** | | | | |
| --- | --- | --- | --- | --- |
| **Scale Group** | **Scale** | **Completed/ provided by?** | **Time (minutes)** | **Method of administration** |
| **Invitation/ Consenting** | **Study invitation/ information letter** | Participant | N/A | Remote (e.g., online, telephone, post) or in person |
|  | **Participant Information Sheet** | Participant | N/A | Remote (e.g., online, telephone, post) or in person |
|  | **Consent Form** | Participant | N/A | Remote (e.g., online, telephone, post) or in person |
| **Screening/ enrolment** | **Screening/ Eligibility Form** | Clinical research team | 10-20 | Telephone or in person |
|  | **Brief Demographics Survey** | Participant | 5-10 | Remote (e.g., online, telephone, post) or in person |
|  | **GAD-7** | Participant | 5-10 | Remote (e.g., online, telephone, post) or in person |
| 1. **Baseline/ randomisation (Week 2 +/-2)** | | | | |
| **Scale Group** | **Scale** | **Completed/ provided by?** | **Time (minutes)** | **Method of administration** |
| **Baseline assessment – primary** |  |  |  |  |
|  | **HADS** | Participant | 5-10 | Remote (e.g., online, telephone, post) or in person |
| **Baseline assessment – secondary** | **Goal Attainment Scaling** | Participant | 20-30 | Remote (e.g., online, telephone, post) or in person |
|  | **Medication/ service use survey** | Participant | 5-10 | Remote (e.g., online, telephone, post) or in person |
| **Baseline assessment - covariate** | **Autistic traits (ARI, CAT-I)** | Participant | 5-10 | Remote (e.g., online, telephone, post) or in person |
| 1. **Intervention period – Part One (Weeks 2-15 +/-2)** | | | | |
| **Scale Group** | **Scale** | **Completed/ provided by?** | **Time (minutes)** | **Method of administration** |
| **App (active vs. comparator) administered** | | Clinical research team | 10-20 | Remote (e.g., online, telephone, post) or in person |
| **App usage** | | Participant | 5-10 daily | Remote (SmartPhone/ Device) |
| **Week-8 remote contact (check in)** | | Clinical research team | 10-20 | Telephone |
| 1. **Endpoint assessment (Week 15 +/-2)** | | | | |
| **Scale Group** | **Scale** | **Completed/ provided by?** | **Time (minutes)** | **Method of administration** |
| **Endpoint assessment – primary** | **App Acceptability Survey/ Interview** | Participant | 30-60 | Remote (e.g., online, telephone, post) or in person |
|  | **GAD-7** | Participant | 5-10 | Remote (e.g., online, telephone, post) or in person |
|  | **HADS** | Participant | 5-10 | Remote (e.g., online, telephone, post) or in person |
| **Endpoint assessment – secondary** | **Goal Attainment Scaling** | Participant | 20-30 | Remote (e.g., online, telephone, post) or in person |
|  | **Medication/ service use survey** | Participant | 5-10 | Remote (e.g., online, telephone, post) or in person |
| **Baseline assessment - covariate** | **Autistic traits (ARI, CAT-I)** | Participant | 5-10 | Remote (e.g., online, telepgone, post) or in person |
| 1. **Follow-up assessment 1 (Week 24 +/-4)** | | | | |
| **Scale Group** | **Scale** | **Completed/ provided by?** | **Time (minutes)** | **Method of administration** |
| **Follow up assessment – primary** | **GAD-7** | Participant | 5-10 | Remote (e.g., online, telephone, post) or in person |
|  | **HADS** | Participant | 5-10 | Remote (e.g., online, telephone, post) or in person |
| **Follow up assessment – secondary** | **Goal Attainment Scaling** | Participant | 20-30 | Remote (e.g., online, telephone, post) or in person |
|  | **Medication/ service use survey** | Participant | 5-10 | Remote (e.g., online, telephone, post) or in person |
| 1. **Follow-up assessment 2 (Week 32 +/-2)** | | | | |
| **Scale Group** | **Scale** | **Completed/ provided by?** | **Time (minutes)** | **Method of administration** |
| **Follow up assessment – primary** | **GAD-7** | Participant | 5-10 | Remote (e.g., online, telephone, post) or in person |
|  | **HADS** | Participant | 5-10 | Remote (e.g., online, telephone, post) or in person |
| **Follow up assessment – secondary** | **Goal Attainment Scaling** | Participant | 20-30 | Remote (e.g., online, telephone, post) or in person |
|  | **Medication/ service use survey** | Participant | 5-10 | Remote (e.g., online, telephone, post) or in person |
| 1. **Follow-up assessment 3 (Week 41 +/-2)** | | | | |
| **Scale Group** | **Scale** | **Completed/ provided by?** | **Time (minutes)** | **Method of administration** |
| **Follow up assessment – primary** | **GAD-7** | Participant | 5-10 | Remote (e.g., online, telephone, post) or in person |
|  | **HADS** | Participant | 5-10 | Remote (e.g., online, telephone, post) or in person |
| **Follow up assessment – secondary** | **Goal Attainment Scaling** | Participant | 20-30 | Remote (e.g., online, telephone, post) or in person |
|  | **Medication/ service use survey** | Participant | 5-10 | Remote (e.g., online, telephone, post) or in person |

1. **Assessment of Effectiveness**

The primary aim of the current study is to assess the acceptability and feasibility of an adapted app-based anxiety intervention for use by autistic people in a real-world clinical setting, rather than intervention effectiveness (which can be further established in a later, full-scale RCT informed by this study).

To assess when and how participants are utilising Molehill Mountain (Objective One), we will seek participant consent to analyse app usage data (i.e., how often participants log in, when, and for how long they use the app each time). We will further establish app acceptability, feasibility and useability (Objective One) via an in-depth post-study app use experience survey (all participants) and semi-structured qualitative interview on app use experience (subsample of participants, who consent).

Both the survey and semi-structured interview format will be tailored, according to whether: a) the participant was offered the app but declined, to establish reasons for decline; b) the participant started the app but stopped using it after only a brief time, to establish reasons for loss of engagement; c) the participant started and continued to use the app, to establish reasons for continued engagement. In scenario c, we will also explore potential differences in app usage between those who use the app and do vs. do not experience an improvement in anxiety symptom severity over the course of the study, to identify whether this may result from differences in frequency/ duration of use and/ or self-reported ability to use the suggested anxiety management strategies incorporated across the different app components described above.

Key indices incorporated in post-study app use experience surveys/ interviews will include: 1) the preparedness of participants to be randomised in a future trial; 2) ease of app administration and use; 3) preferred app features, content, and structure; 4) non-preferred app features, content, and structure; 5) experiences of app technical functionality; and 6) subjective reflections on the impact of app use on anxiety and everyday wellbeing/ functioning.

Nevertheless, the impact of the active app-based anxiety intervention on anxiety symptom severity (primary endpoint) and broader functioning according to Goal Attainment Scaling and medication/ service use (secondary endpoints) will be assessed as additional indices of effectiveness. Minimally clinically relevant change in anxiety symptom severity, as assessed using the GAD-7/ HADS, will be indicated by a reduction of ≥0.5 standard deviations from baseline to endpoint assessment (please also see Norman, Sloan and Wyrwich, 2003), as well as each follow-up assessment since the optimal timeline for outcome measurement has not yet been established (and that we aim to identify in the current study).

1. **Assessment of Safety**
   1. ***Specification, Timing and Recording of Safety Parameters***

Safety monitoring will take place continuously throughout the trial (i.e., from participant consent to the end of trial proper; please also see Section 10.6) and at each participant contact. Participants will be provided with the contact details of the research team and can therefore report AE/ SAE at any time. The research team will also monitor specific participant responses at baseline, endpoint and follow-up assessments (e.g., reports of suicidal intent). Participants will be made aware in the Information and Consent Forms that in cases where there is sufficient evidence to raise serious concern about their safety there are legal obligations that may override their confidentiality and they may be encouraged by the clinical research team to seek support. In case of disclosures of suicidal intent, we have established mechanisms to support participants (e.g., immediate referral to KCH A&E where there is a plan of ‘how’ and ‘when’). In case of other disclosures (safeguarding) the research team will notify the Chief Investigator, as the lead on-call clinician providing study oversight, who will then initiate the correct procedure following local guidelines. The Week 8 check-in (please see Table 1) is also designed to monitor and record any possible participant concerns, AE and SAE using telephone or in-person contact, in case these have gone undetected in remote contact.

- 1. ***Procedures for Recording and Reporting Adverse Events***

| **Adverse Event (AE):** Any untoward medical occurrence in a participant to whom a therapy has been administered, including occurrences that are not necessarily caused by or related to that therapy.  **Adverse Reaction (AR):** Any untoward and unintended response in a participant to a therapy that is related to any duration of therapy administered to that participant.  **Unexpected Adverse Reaction** **(UAR)**: An adverse reaction the nature and severity of which is not consistent with the information known about the therapy in question in the view of the Investigator.  **In non-CTIMPs, a serious adverse event (SAE) is defined as an untoward occurrence that:**  (a) results in death;  (b) is life-threatening;  (c) requires hospitalisation or prolongation of existing hospitalisation;  (d) results in persistent or significant disability or incapacity;  (e) consists of a congenital anomaly or birth defect; or  (f) is otherwise considered medically significant by the Investigator**.**  **An SAE occurring to a research participant will be reported to the main REC, where in the opinion of the Chief Investigator (CI) the event was:**   - Related – that is, it resulted from administration of any of the research procedures, and - Unexpected – that is, the type of event is not listed in the protocol as an expected occurrence.   *Reporting Responsibilities*  All AEs/ ARs/ UARs will be clearly recorded in the participant CRF providing as much detail as possible. Details will also and included in the annual progress report for the project.  All SAEs, SARs and SUSARs (excepting those specified in this protocol as not requiring reporting) will be reported immediately to the Chief Investigator and to the Sponsor.  Reports of SAEs that are:   - **related**to the study (i.e., they resulted from administration of any of the research procedures) and - **unexpected**(i.e., not listed in the protocol as an expected occurrence)   will be submitted to the REC using the [Non-CTIMP safety report to REC form](https://www.hra.nhs.uk/documents/1087/safety-report-form-non-ctimp.docx). These will be sent within 15 days of the CI becoming aware of the event.  **Table 3. *Procedures for recording and reporting (S)AEs.*** | | | | | |
| --- | --- | --- | --- | --- | --- |
|  | **Who** | **When** | **How** | **To Whom** |  |
| **SAE** | Chief Investigator | Within 15 days of CI becoming aware of the event | SAE Report form for Non-CTIMPs, available from NRES website | Main REC with a copy to the sponsor |  |
| **Urgent Safety Measures** | Chief Investigator | Immediately  Within 3 days | By phone  Notice in writing setting out reasons for the urgent safety measures and the plan for future action. | Main REC  Main REC with a copy sent to the sponsor. The Main REC will acknowledge this within 30 days of receipt. |  |
| **Progress Reports** | Chief Investigator | Annually (starting 12 months after the date of favourable opinion) | Annual Progress Report Form (non-CTIMPs) available from the NRES website | Main REC with a copy to the sponsor |  |
| **Declaration of the conclusion or early termination of the study** | Chief Investigator | Within 90 days (conclusion)  Within 15 days (early termination)  *The end of study should be defined in the protocol* | End of Study Declaration form available from the NRES website | Main REC with a copy to the sponsor |  |
| **Summary of final Report** | Chief Investigator | Within one year of conclusion of the Research | No Standard Format  However, the following Information should be included:-  Where the study has met its objectives, the main findings and arrangements for publication or dissemination including feedback to subjects | Main REC with a copy to be sent to the sponsor |  |

- - 1. *Adverse events that do not require reporting*
- Features of the condition(s) being studied and expected signs of progression/ fluctuation (including increases in severity, so long as not continuous) in the features of the condition(s) being studied, unless they are deemed by the Investigator to be more severe than expected for the participant.
- Situations where an untoward medical occurrence did not occur (e.g., contact with health/ social care services due to social factors).
- Medical or surgical procedures: it is the condition that leads to the procedure that constitutes an AE.
  1. ***Stopping Rules***

The trial may be prematurely discontinued by the Sponsor or CI on the basis of new safety information or for other reasons given by the Trial Management Group or ethics committee concerned.

The trial may also be prematurely discontinued due to lack of recruitment or upon advice from a Trial Management Group, who will make a recommendation to the sponsor. If the study is prematurely discontinued, active participants will be informed and no further participant data will be collected.

1. **Statistics**
   1. ***Sample Size***

The proposed sample size will provide sufficient power of 0.8 to detect moderate effects of intervention (f 2 ≥0.15) at p<0.05, while accounting for intervention frequency and duration, baseline anxiety severity, age, sex and psychotropic medication use; in addition to being a feasible sample to recruit in a 12-month period, based on current estimates of available target participant pools.

This feasibility trial will also support a formal sample size calculation for a future full-scale RCT.

- 1. ***Analysis***

Analysis will be informed by the Consolidated Standards of Reporting Trials (CONSORT) guidelines. Specifically, details of participant flow and recruitment will be reported, including the number of participants declined/ enrolled, receiving the intervention, analysed on primary outcome(s), and losses/ exclusions. Important AEs will also be reported in an anonymised fashion.

Descriptive statistics on participant demographic/ clinical characteristics at baseline, medication/ service use at endpoint and follow-ups, and objective app use data; as well as subjective experiences of app use via the app acceptability survey, will be reported.

Thematic analysis will be performed on semi-structured qualitative interviews of app use experience, using Braun and Clarke’s (2006) six-step method. Interviews will be conducted with a subsample of participants – initially 10 participants from each subgroup detailed above (a) participant declined app; b) participant stopped app after brief time; c) participant continued with app, anxiety improved; d) participant continued with app, anxiety did not improve). If data saturation has not been reached following recruitment of these 40 participants, interviews will continue in multiples of 3 per subgroups until such time saturation is reach, or the sample size for that subgroup exceeds the maximally optimal figure of approximately 30 – based on literature advising on the optimal sample size for meaningful qualitative research.

Finally, we will assess the performance of our primary outcome measures (GAD-7/ HADS), including establishing the optimal timing for endpoint/ follow-up assessments, and a threshold for clinically meaningful reduction in anxiety through exploring relationships between the GAD-7/ HADS with our secondary outcome measures (Goal Attainment Scaling, medication/ service use) and semi-structured interviews. Relationships between demographic factors (e.g., age, sex) and app usage/ outcomes will be investigated to ascertain whether any additional covariates should be included in core analyses and/ or whether subgroup analyses should be performed.

Deviations from the original statistical analysis plan will be reported as exploratory, rather than pre-defined, and will be reported with a full justification.

1. **Trial Management Group**

The Trial Management Group (TMG) will monitor and evaluate the progress, workings/ management and potential AEs, meeting on at least a 2-monthly basis, while providing reports of AEs on a quarterly basis. The TMG will consist of the following members:

- Professor Declan Murphy – Clinical research expert advisor
- Professor Emily Simonoff – Clinical research expert advisor and Molehill Mountain app advisor
- Dr Bethany Oakley – Scientific coordinator (trial administration/ management/ analytics/ reporting)
- Dr Rachel Kent – Clinical expert advisor and Molehill Mountain app advisor
- Dr Ann Ozsivadjian – Clinical expert advisor and Molehill Mountain app advisor
- Dr Sophie Doswell – Clinical expert advisor and clinical trial coordinator (recruitment/ monitoring)
- Dr Antonia Dittner - Clinical expert advisor and clinical trial coordinator (recruitment/ monitoring)
- Mr Andy Clarke – Molehill Mountain Product Owner
- Stakeholder group
- Named research team members (Research Assistant, students)

Meeting agenda, minutes and action points will be recorded in writing by the Scientific Coordinator, with a signed copy of each stored electronically and, in the case of meeting minutes, sent to the sponsor for oversight.

1. **Direct Access to Source Data and Documents**

The Investigator(s) will permit trial-related monitoring, audits and REC review by providing the Sponsor(s), and REC direct access to source data and other documents (e.g., participant CRF). Participants will be informed of this in the Information and Consent Forms.

1. **Ethics & Regulatory Approvals**

The trial will be conducted in compliance with the principles of the Declaration of Helsinki (1996), the principles of Good Clinical Practice (GCP) and in accordance with all applicable regulatory requirements, including but not limited to, the UK policy framework for health and social care research and the Mental Capacity Act 2005.

This protocol and related documents will be submitted for review to London – Queen Square Research Ethics Committee (REC).

The Chief Investigator will submit a final report at conclusion of the trial to the funder, the REC and the Sponsor.

1. **Quality Assurance**

Monitoring of this trial will be to ensure compliance with GCP standards and scientific integrity will be managed by the study team, with oversight from the Trial Management Group; which includes several members with significant experience in clinical trials.

Data quality will be recorded in the participant electronic CRF (eCRF), stored locally on a password protected computer. Trial data will be quality control checked by the research team against Standard Operating Procedures (SOPs) and scoring manuals for each measure. In any cases where data are scored manually (e.g., paper-and-pencil questionnaires), the research team will double code and double enter the data.

Throughout the study, the research team will hold regular teleconferences to monitor recruitment and assessment progress and to review eCRF entries, in the context of the Trial Management Committee (please see above).

1. **Data Handling and Management**

The Chief Investigator will act as custodian for the trial data. The following guidelines will be strictly adhered to:

- Participant data will be pseudononymised (i.e., identifying information like names are replaced by a unique code).
- All pseudononymised data will be stored on a secure, password protected computer (i.e., GDPR compliant workplace desktop/ laptop, secure workplace server).
- All trial data will be stored in line with the Data Protection Act and archived in line with Sponsor requirements*.*
- Paper files (e.g., consent form) that may contain identifying information will be stored in a locked cabinet, in a locked office with access by the immediate research team only.

The research team will maintain adequate and accurate records to enable the conduct of the study to be fully reconstructed, including but not limited to, the protocol/ amendment, consent forms, eCRFs, outcome measures, ethical review documents and correspondence, TMG meetings and all source documents.

1. **Publication Policy**

It is intended that the results of the study will be reported and disseminated at international conferences and in peer-reviewed scientific journals. Summary information regarding the trial (e.g., protocol, main results) may also be required to be posted in clinical trial registries and/ or open science repositories. Participants will be made aware of this in the Information and Consent Forms.

1. **Insurance / Indemnity**

King’s College London and NHS indemnity will apply. The lead sponsor, King's College London, will take primary responsibility for ensuring that the design of the study meets appropriate standards and that arrangements are in place to ensure appropriate conduct and reporting. King's College London also provides cover under its No Fault Compensation Insurance, which provides for payment of damages or compensation in respect of any claim made by a research subject for bodily injury arising out of participation in a clinical trial or healthy volunteer study (with certain restrictions). The co-sponsor, South London and Maudsley NHS Foundation Trust, take responsibility for arranging the initiation and management of this research, and will take responsibility for ensuring that appropriate standards, conduct and reporting are adhered to regarding its facilities and staff involved with the project.

1. **References**

American Psychiatric Association. (2013). *Diagnostic and Statistical Manual - 5th Edition*. Arlington, VA: American Psychiatric Association Publishing.

Autistica. (2017). Research Strategy 2017 - 2021. Retrieved from https://www.autistica.org.uk/downloads/files/Autistica-Research-Strategy-2017-2021.pdf

Benevides, T. W., Shore, S. M., Andresen, M.-L., Caplan, R., Cook, B., Gassner, D. L., … Wittig, K. (2020). Interventions to address health outcomes among autistic adults: A systematic review. *Autism*, 1362361320913664. https://doi.org/10.1177/1362361320913664

Cooper, K., Loades, M. E., & Russell, A. (2018). Adapting psychological therapies for autism. *Research in Autism Spectrum Disorders*, *45*, 43–50. https://doi.org/https://doi.org/10.1016/j.rasd.2017.11.002

Excellence, N. I. of H. and C. (2016). *Autism spectrum disorder in adults : diagnosis and management*.

Hollocks, M. J., Lerh, J. W., Magiati, I., Meiser-Stedman, R., & Brugha, T. S. (2019). Anxiety and depression in adults with autism spectrum disorder: a systematic review and meta-analysis. *Psychological Medicine*, *49*(4), 559–572. https://doi.org/DOI: 10.1017/S0033291718002283

Maenner, Shaw, K, A., Baio, J., Washington, A., Patrick, M., DiRienzo, M., … Dietz. (2020). *Prevalence of Autism Spectrum Disorder Among Children Aged 8 Years — Autism and Developmental Disabilities Monitoring Network, 11 Sites, United States, 2016.* (No. 69 (4)). Retrieved from https://www.cdc.gov/mmwr/volumes/69/ss/ss6904a1.htm?s_cid=ss6904a1_w

NHS England. (2019). *The NHS Long Term Plan*. Retrieved from https://www.longtermplan.nhs.uk/wp-content/uploads/2019/08/nhs-long-term-plan-version-1.2.pdf

Norman, G. R., Sloan, J. A., & Wyrwich, K. W. (2003). Interpretation of changes in health-related quality of life: the remarkable universality of half a standard deviation. *Medical Care*, *41*(5), 582–592. https://doi.org/10.1097/01.MLR.0000062554.74615.4C

Oakley, B., Tillmann, J., Ahmad, J., Crawley, D., San Jose Caceres, A., Holt, R., … Loth, E. (2020). How do core autism traits and associated symptoms relate to quality of life? Findings from the Longitudinal European Autism Project. *Autism*, *25*(2).

Rosenberger, W. F., Sverdlov, O., & Hu, F. (2012). Adaptive randomization for clinical trials. *Journal of Biopharmaceutical Statistics*, *22*(4), 719–736. https://doi.org/10.1080/10543406.2012.676535

Simonoff, E., Pickles, A., Charman, T., Chandler, S., Loucas, T., & Baird, G. (2008). Psychiatric disorders in children with autism spectrum disorders: Prevalence, comorbidity, and associated factors in a population-derived sample. *Journal of the American Academy of Child and Adolescent Psychiatry*, *47*(8), 921–929. https://doi.org/10.1097/CHI.0b013e318179964f

Sutherland, R., Trembath, D., & Roberts, J. (2018). Telehealth and autism: A systematic search and review of the literature. *International Journal of Speech-Language Pathology*, *20*(3), 324–336. https://doi.org/10.1080/17549507.2018.1465123

van Steensel, F. J. A., Bögels, S. M., & Perrin, S. (2011). Anxiety Disorders in Children and Adolescents with Autistic Spectrum Disorders: A Meta-Analysis. *Clinical Child and Family Psychology Review*, *14*(3), 302–317. https://doi.org/10.1007/s10567-011-0097-0

White, S. W., Simmons, G. L., Gotham, K. O., Conner, C. M., Smith, I. C., Beck, K. B., & Mazefsky, C. A. (2018). Psychosocial Treatments Targeting Anxiety and Depression in Adolescents and Adults on the Autism Spectrum: Review of the Latest Research and Recommended Future Directions. *Current Psychiatry Reports*, *20*(10), 82. https://doi.org/10.1007/s11920-018-0949-0

Williams K, B. A. R. M. S. N., & Hazell, P. (2013). Selective serotonin reuptake inhibitors (SSRIs) for autism spectrum disorders (ASD). *Cochrane Database of Systematic Reviews*, (8). https://doi.org/10.1002/14651858.CD004677.pub3

1. **Appendixes**

*This section should contain all pertinent documents associated with the management of the study. The following lists a few examples of potential attachments:*

- Sample Information Sheet
- Sample Consent Form
- Study SOPs

Please see: [Molehill Mountain Feasibility Study. - Full Text View - ClinicalTrials.gov](https://clinicaltrials.gov/ct2/show/NCT05302167)
