# Supplementary material for: Molehill Mountain feasibility study: Protocol for a non-randomised pilot trial of a novel app-based anxiety intervention for autistic people
Source: PLoS One. 2023 Jul 5;18(7):e0286792. doi: 10.1371/journal.pone.0286792 (PMC10321642; doi:10.1371/journal.pone.0286792)
Supplement: Attachment — Submitted filename: Response to Reviewers.docx [file pone.0286792.s003.docx]

23/03/2023

Re: Manuscript Resubmission – Study Protocol

Dear Reviewers and Editorial team,

Thank you for your invitation to submit a revised version of our manuscript, titled “*Molehill Mountain Feasibility Study: Protocol for a non-randomised pilot trial of a novel app-based anxiety intervention for autistic people*” (PONE-D-22-34959), for publication in PLOS ONE.

We would like to first take this opportunity to thank the reviewers and editorial team for their time and careful consideration of our manuscript and we are pleased that the topic and approach of our work was well received. The comments and suggestions made have been highly valuable and we feel that, having implemented these, our manuscript is much improved and a strong candidate for publication in PLOS ONE. In particular, we have now provided far more detail on the study design and method to ensure that the study approaches and order of events are clearer for the reader.

Please find a point-by-point response to comments from the editorial team and reviewers below, with our responses shown in italics.

We thank you again for considering our work and we look forward to hearing from you in the near future.

Yours Sincerely, and on behalf of all co-authors,

Dr. Bethany Oakley

**Response to Journal Requirements**

1. Please ensure that your manuscript meets PLOS ONE's style requirements, including those for file naming. The PLOS ONE style templates can be found at
   [https://journals](https://journals/).plos.org/plosone/s/file?id=wjVg/PLOSOne_formatting_sample_main_body.pdf and 
   <https://journals.plos.org/plosone/s/file?id=ba62/PLOSOne_formatting_sample_title_authors_affiliations.pdf>

*We apologise that some of our manuscript style/ formatting did not initially align with PLOS ONE requirements. We have now compared our manuscript against the PLOS ONE style templates and made the following changes:*

- *Updating the file names for resubmission*
- *Provision of short title*
- *Indicating group authorship and providing author contributions on title pages*
- *Changes to heading format (Level 1-3)*
- *Including Figure headings/ captions in main text*
- *Moving position of acknowledgments*
- *Adding supporting information section after references*
- *Removing funding/ competing interests and data availability sections from main text*

2. We note that the grant information you provided in the ‘Funding Information’ and ‘Financial Disclosure’ sections do not match.

When you resubmit, please ensure that you provide the correct grant numbers for the awards you received for your study in the ‘Funding Information’ section.

*Thank you for flagging this. We have now updated the Funding Information section accordingly.*

3. Thank you for stating the following in the Funding Section of your manuscript:

"This study received funding from the MRC Confidence in Concept award 2019 (1118148). Dr Oakley, Professor Simonoff and Professor Murphy report grants during the conduct of (but unrelated to) this study from the Innovative Medicines Initiative 2 Joint Undertaking under grant agreement No 777394 for the project AIMS-2-TRIALS. This Joint Undertaking receives support from the European Union’s Horizon 2020 research and innovation programme and EFPIA and SFARI, Autistica, Autism Speaks. The views expressed are those of the author(s) and not necessarily those of the NHS, MRC, nor IMI 2JU. The funders had no role in the conceptualisation of this study, nor the development of this publication."

We note that you have provided funding information that is not currently declared in your Funding Statement. However, funding information should not appear in the Acknowledgments section or other areas of your manuscript. We will only publish funding information present in the Funding Statement section of the online submission form.

Please remove any funding-related text from the manuscript and let us know how you would like to update your Funding Statement. Currently, your Funding Statement reads as follows:

"This study received funding from the MRC Confidence in Concept award 2019 (1118148) - awarded to BO, ES. Dr Oakley, Professor Simonoff and Professor Murphy report grants during the conduct of (but unrelated to) this study from the Innovative Medicines Initiative 2 Joint Undertaking under grant agreement No 777394 for the project AIMS-2-TRIALS. This Joint Undertaking receives support from the European Union’s Horizon 2020 research and innovation programme and EFPIA and SFARI, Autistica, Autism Speaks. The views expressed are those of the author(s) and not necessarily those of the NHS, MRC, nor IMI 2JU. The funders had no role in the conceptualisation of this study, nor the development of this publication. "

Please include your amended statements within your cover letter; we will change the online submission form on your behalf.

*Following journal requirements 1 and 2 (above), we have firstly removed any funding related text from the manuscript. We have also updated the Funding Information section to match the Financial Disclosures text. We wish to maintain the current Funding Statement, with some minor edits, as written below:*

*“This study received funding from the MRC Confidence in Concept award 2019 (1118148) - awarded to BO, ES. DM, ES, BO report grants during the conduct of (but unrelated to) this study from the Innovative Medicines Initiative 2 Joint Undertaking under grant agreement No 777394 for the project AIMS-2-TRIALS. This Joint Undertaking receives support from the European Union’s Horizon 2020 research and innovation programme and EFPIA and SFARI, Autistica, Autism Speaks. The views expressed are those of the author(s) and not necessarily those of the NHS, MRC, nor IHI-JU2. The funders had no role in the conceptualisation of this study, nor the development of this publication.”*

4. In your Data Availability statement, you have not specified where the minimal data set underlying the results described in your manuscript can be found. PLOS defines a study's minimal data set as the underlying data used to reach the conclusions drawn in the manuscript and any additional data required to replicate the reported study findings in their entirety. All PLOS journals require that the minimal data set be made fully available. For more information about our data policy, please see [http://journals](http://journals/).plos.org/plosone/s/data-availability.

Upon re-submitting your revised manuscript, please upload your study’s minimal underlying data set as either Supporting Information files or to a stable, public repository and include the relevant URLs, DOIs, or accession numbers within your revised cover letter. For a list of acceptable repositories, please see [http://journals](http://journals/).plos.org/plosone/s/data-availability#loc-recommended-repositories. Any potentially identifying patient information must be fully anonymized.

Important: If there are ethical or legal restrictions to sharing your data publicly, please explain these restrictions in detail. Please see our guidelines for more information on what we consider unacceptable restrictions to publicly sharing data: [http://journals](http://journals/).plos.org/plosone/s/data-availability#loc-unacceptable-data-access-restrictions. Note that it is not acceptable for the authors to be the sole named individuals responsible for ensuring data access.

We will update your Data Availability statement to reflect the information you provide in your cover letter.

*We are committed to making the minimal dataset for this study publicly available, which is also a requirement of our funder. However, as this is a study protocol, we have not yet completed data collection and therefore cannot upload the dataset at this time (neither to the journal, nor repository).*

*A fully anonymised dataset from this study will be made publicly available after data collection has ended via the clinical trials registry/ open data repository, for those participants who consent to this.*

*Therefore, as required in the submission portal, we have updated our data availability statement to: “No datasets were generated or analysed during the current study. All relevant data from this study will be made available upon study completion.”*

5. Please amend the manuscript submission data (via Edit Submission) to include author Judd, Adrian., Baldoza, Saffron., Hearn, Amy.

One of the noted authors is a group or consortium The Molehill Mountain Advisory Group. In addition to naming the author group, please list the individual authors and affiliations within this group in the acknowledgments section of your manuscript. Please also indicate clearly a lead author for this group along with a contact email address.

*We have now added the three additional co-authors to the Manuscript Submission Data, as we received their consent to provide their personal email addresses.*

*Regarding group authorship, please see Acknowledgements section for individual authors (four members of the group did not provide their consent to be named individually).*

*As this is a lived experience expertise group (rather than a consortia), we have indicated the group affiliation as King’s College London, where the group was formed and is hosted, with correspondence to be directed to the lead author of the manuscript whose contact details now clearly stated in the Acknowledgements section.*

**Response to Editor Comments**

1. Introduction
1) Please elaborate on the main sources/reasons of anxiety in individuals with autism.

*We agree that the candidate causal/ maintenance factors associated with anxiety in autism are important to address, given that several of these factors should represent key targets for intervention to reduce anxiety.*

*Further evidence is required to elucidate the underpinning mechanisms for anxiety in autism and how they overlap and/or are distinct from those identified in non-autistic populations (acknowledged in new brief addition in brackets on Page 7, Line 118). Nevertheless, existing research would suggest that these mechanisms are multifaceted and include not only genetic/ neurobiological (e.g., autonomic dysregulation due to chronic stress – beyond the scope of this paper, but briefly now referenced on Page 6, Line 98), but also social/ environmental factors (e.g., stigma, social and sensory environmental pressures).*

*In our resubmitted manuscript, we have particularly focused on adding an overview of factors implicated in anxiety in autism that are also incorporated into the app-based intervention used for this study (and as potentially useful additions to CBT and related approaches more broadly) and thus directly linked to the current research. These include, sensory processing differences, alexithymia (difficulties identifying/ describing emotions), emotion dysregulation, and cognitive biases (e.g., intolerance of uncertainty, reduced cognitive flexibility; please see Page 6, Lines 103-108) – experienced at higher rates in autistic, as compared to non-autistic, populations.*

2. Materials and methods
1) The sample size calculation is unclear and there is no reference support. Please explain in details.

*As our study is a feasibility study, we based our original sample size on practicality/ feasibility for a 12-month recruitment window, and our best estimate of the available target participant pool (i.e., those on the waitlist) in local mental health services – following guidance on the sample size for pilot study designs as in Leon et al (2011; 10.1016/j.jpsychires.2010.10.008). This was to allow us to address key study feasibility targets, such as proportion who are eligible and consent, proportion who use the app over 13-weeks and complete outcome measures, ease of app administration and use, preferred and non-preferred app features, content, and structure, subjective reflections on the impact of app use on anxiety and everyday wellbeing/ functioning, and preparedness of participants to be randomised in a future trial.*

*Following the recommendations of the Editors and Reviewers, we now also provide an indicative sample size calculation to further support our approach. We have updated this in the manuscript accordingly (Page 9, Line 175-185).*

*Reflecting on the combination of guidance on pilot study sample size estimates, and the power calculation provided, we have revised our target sample size to approximately 100 participants.*

3. 2) Like 158: How to determine mild to severe anxiety?

*We have now specified that we establish this inclusion criteria as indicated by a score of at least 5 or above on the GAD-7 during screening (please see Page 10, Line 191-192) and we provide specifiers for all bands (mild, moderate severe) of the GAD-7 scoring cutoffs (please see Page 13, Lines 273-274) to provide more context on this use of the tool for the reader.*

3. 3) Please elaborate on the content and technical aspects of the mobile application (? password required/ Andriod or IOS/ layout).

*We agree that it would be helpful to the reader to provide some more detail on the content and technical aspects of the app. We now confirm in the main text of the manuscript that the app is compatible with both iOS and Android (please see Page 11, Line 228), that the app is accessed through a password protected individual user account/ profile (please see Page 12, Lines 257-258), that participants can adjust their privacy and sharing preferences directly in the app under Settings (please see Page 15, Lines 315-316), and more detail about the daily ‘Check In’ function (please see Page 12, Lines 257-260).*

*Further technical details and development information about the app are provided on the Autistica website (*[*Molehill Mountain | Autistica*](https://www.autistica.org.uk/molehill-mountain)*), which we also reference in the manuscript.*

4. 4) Please detail how the outcome measures will be evaluated (? In-app survey).

*We clarify this by adding that the baseline/ endpoint and follow up assessments (which incorporate the outcome measures used in this study) are administered by the research team via Qualtrics (please see Page 10, Figure 1 Legend; Page 16 Lines 353-354 and 355-356, 360; Page 17 Line 371), or the participants’ preferred communication method to enhance inclusivity of the study design (e.g., via email, post, over the telephone).*

**Response to Reviewer Comments**

Reviewer #1 Comments and Author Response:

1. Introduction
Line 139 – 141, the statement ‘To address this unmet need, the current paper details the protocol for a feasibility study and single-arm pilot trial of a novel app-based therapeutic approach (‘Molehill Mountain’) that has been developed with’ requires revision and to align with the title.

*We thank the reviewer for flagging this discrepancy and have updated this statement in the Introduction accordingly to align with the title (please see Page 8, Lines 158-159).*

2. Materials and methods
The location to conduct the study, recruitment, and study design employed is to be stated.

*Following the reviewers’ recommendation, we have added a new ‘Design’ section to the Materials and methods (please see Page 9, Lines 169-173), which also details the primary study location. Further details are also provided regarding recruitment locations on Page 9, Lines 186-187.*

3. Sample size calculation
Line 152, the sample size calculation is unclear and requires more information e.g. to include/state clearly alpha 0.05, one-tailed or two-tailed test, the outcome variable used, and include the word effect size.

*We apologise for the lack of clarity on the sample size previously provided and have updated this section in the manuscript accordingly (Page 9, Line 175-185). For a full explanation, please see response to Editor Comments, comment and response #2, above.*

4. Outcome measures
Line 207-244, Line 245-251, Line 250-251, Line 294, Line 309-302, it is not clear how the inventories/questionnaires will be administered. How the inventories/questionnaires (self-report) will be given to subjects and how the participant completed the questionnaires/assessment in the study is to be clearly stated.

*As also noted in response to Editor Comments, comment and response #4, we now clarify this by adding that the baseline/ endpoint and follow up assessments (which incorporate the outcome measures used in this study) are administered by the research team via Qualtrics (please see Page 10, Figure 1 Legend; Page 16 Lines 353-354 and 355-356, 360; Page 17 Line 371), or the participants’ preferred communication method to enhance inclusivity of the study design (e.g., via email, post, over the telephone).*

5. It would be good to provide an outline of the final sample questionnaire/survey looks like when incorporating various questionnaires/questions that the participants will be receiving and indicate how long it will take for the participants to complete the questionnaire altogether.

*We thank the reviewer for the suggestion to provide an indication of how long it will take to complete the survey components of the study and we have now added this information into Figure 1, where we provide a full summary of which questionnaires are included per survey/ timepoint.*

6. Procedure
   Line 273, 278, Line 301, more information on the screening process conducted, how the consent is obtained, how the subjects/participants will be invited and the method of the exit survey will be carried out is to be stated.

*We agree with the reviewer that more details and context would be helpful for the reader in the Procedures section and make the order of events in the study clearer. We therefore provide additional information regarding inviting participants (please see Page 16, Lines 336-338, 340-343), the screening process (please see Page 16, Lines 344-352), obtaining consent (please see Page 16, Lines 352-354), and exit survey administration (please see Page 16, Lines 354-358 and Figure 1 legend).*

7. Data quality and management
   Line 306, full name for CRF to be stated e.g. Case Report Form. The monitoring data quality is to be stated.

*We apologise for this oversight and have now corrected the acronym to the full term (please see Page 18, Line 401) and further detail added to this section (Page 18, Lines 398-401).*

8. Statistical analysis plan
   Line 320, 327, 330, 332, the sentence requires revision.

*We have followed the above recommendation of the reviewer by:*

- *Clarifying that results with be reported according to CONSORT extension for non randomised trial design (Page 19, Lines 418-419).*
- *Clarifying from which timepoints descriptive statistics will be reported on which measures (Page 19, Lines 425-428).*
- *Clarifying that performance of outcome measures will inform the design (e.g., in terms of optimal assessments) for a future RCT (please see Page 428-435) and interpretation of changes in anxiety that may be meaningful (e.g., because they relate to other functional outcomes).*

  A preliminary possible statistical test could be proposed in the analyses.

*We agree with the reviewer that we should be more specific in this regard and have added some relevant examples (please see Page 19, Lines 425-426, 430-431, 437-438).*

Line 344, [50] to be placed after the word method.

*We thank the reviewer for noticing this typo, and have updated accordingly (Page 20, Line 447).*

Line 346, apart from what were mentioned in the statistical analyses plan, information such as the method to conduct semi-structured interviews, the software to run the statistical analysis and qualitative analysis (if any), the level of accepted statistical significance, correction method (if any), missing data/handling, effect size, 95% CI etc to be stated.

*We have added further information on these aspects in the resubmitted manuscript, including our planned approach to statistical reporting and interpretation (please see Page 19, Lines 413-417), the software to be used (please see Page 19, Lines 413-414; Page 20, Line 447). We also provide a more detailed rationale for the app experience interview component on Page 17, Lines 375-388.*

9. Figure 1, it would be good to add the mode of administration and how the assessment is done for those items highlighted and denoted in the footnote e.g. email, in-person, online etc

*We now indicate the mode of administration for each assessment in the Figure legend (please see Page 10), also complementing our edits in response to Editor (comment and response #4) and Reviewer #1 (comment and response #4) queries regarding how the questionnaires are administered.*

Reviewer #2 Comments and Author Response:

1. Participants:
   • The inclusion criteria are noted to include age, be experiencing anxiety, providing consent, English language fluency, and ability to use a mobile phone app, but there is no information regarding diagnostic status. Earlier in the introduction, it was noted that digital tools can be helpful for those waiting to receive a diagnosis or who are self-diagnosed. How is ASD diagnostic status confirmed or is it by self-report? Is participation based on presence of autistic traits, which are known to be commonly observed in the general population of individuals without an ASD diagnosis (please see Sasson & Bottema-Beutel, 2022 "Studies of autistic traits in the general population are not studies of autism")? As recruitment is through public advertising or outpatient clinic services which may include individuals without an ASD diagnosis, how can the results be interpreted or related to autistic individuals? Could it just be that these ASD-specific modifications and digital app presentation are good for CBT in general and beneficial to anyone, not specifically those with ASD if diagnostic status is not confirmed?

*We apologise that we were not clearer on this point and agree with the reviewer that this is an important clarification for us to make. We confirm that we do require participants to have an existing autism diagnosis to take part in the study. We had previously included reference to an existing diagnosis of autism along with information on the recruitment approach, which meant that this inclusion criteria was not obvious in our original submission. Therefore, we have now stated as part of the inclusion criteria that an autism diagnosis is required to take part in this study (please see Page 9, Line 188).*

*We ascertain this information in two ways. For participants recruited through clinic services, the clinic team screen for potentially eligible participants and thus do not include those without an existing autism diagnosis (as per existing clinic records) in this process. For all participants, and particularly relevant to those recruited outside of clinic services, the research team also request confirmation of autism diagnosis (including the specific diagnosis if known e.g., ASD, Asperger’s, and who gave this diagnosis) during the screening process and we have added this information in brief to Page 16, Line 350-351.*

*In terms of the later interpretation of findings, we also include two dimensional measures of autistic traits in our assessment battery (CAT-I, ARI) to enable us to ascertain whether baseline autistic traits influence app usage/ its impact on anxiety outcomes across the whole sample to identify potential individual variation in app acceptability/ effectiveness in autism.*

2. While the exclusion criteria include things such as recent participation in CBT and recent psychotropic medication use at the start of the study, is there any data being collected from participants as they may start therapy or medication (or other supportive services) while using this app as that may also have an impact on interpretation of results?

*We thank the reviewer for highlighting that we had inadvertently not provided details of our questionnaire on medication/ service use (secondary outcome measure) in the methods, which we now provide on Page 14, Lines 299-305.*

3. Intervention:
   • I was unable to find the “evidence-based adapted CBT principles” on the website provided. Please include those references in this manuscript as they provide the critical foundation upon which this app and its specific application for autistic individuals rests and is not provided. It is difficult to evaluate the appropriateness of the modifications and specificity to this population without knowing which evidence-based CBT principles are included in the app or the ASD-specific modifications (and support for them – unless you are referring to those in references 26-29). CBT may have many elements and it is difficult to know which ones are included in the app to evaluate the data and intervention effectiveness.

*We agree with the reviewer that the evidence-base for the features of the app are core to the study approach and interpretation. We have therefore added further detail about specific app components and their underlying evidence base on Page 12, Lines 248-255 and provide additional references, including from our team, to support this (please see Chew, Ozsivadjian, Hollocks and Magiati, 2022; Wood et al, 2020).*
